# Role of Platelet-Activating Factor in Necrotizing Enterocolitis in Pediatric Patients

**DOI:** 10.7759/cureus.90671

**Published:** 2025-08-21

**Authors:** Hinal Shah, Kush Patel, Joelle Robinson, Andrew Eldeiry, Amber Khan, Victor Flores, Ramon Sison, Christian Hietanen

**Affiliations:** 1 Pediatric Gastroenterology, Touro College of Osteopathic Medicine, Middletown, USA; 2 Primary Care, Touro College of Osteopathic Medicine, Middletown, USA

**Keywords:** breast milk, intestinal barrier, necrotizing enterocolitis, platelet-activating factor, prematurity

## Abstract

Necrotizing enterocolitis (NEC) is a serious gastrointestinal disease primarily affecting premature and very-low-birth-weight infants, with an incidence of 1-7% among NICU admissions and up to 10% in infants weighing less than 1500 grams. Clinical presentation often includes feeding intolerance, abdominal distension, bloody stools, and signs of systemic illness such as temperature instability, apnea, and lethargy. Mortality rates remain high, especially in severe or surgically managed cases, underscoring the need for early recognition and intervention. The development of NEC is influenced by a range of factors, including gestational age, birth weight, chorioamnionitis, and genetic predisposition, all of which have been previously explored, shedding light on the multifactorial nature of NEC initiation. Platelet-activating factor (PAF), a proinflammatory phospholipid, plays a crucial role in diverse physiological and pathological processes, influencing cellular functions, apoptosis, inflammation, and wound healing through de novo and remodeling pathways. Dysregulation of PAF is implicated in various diseases, including NEC. This review will explore the role of PAF throughout the development and progression of NEC in pediatric patients, the pathways activated by PAF, its impact on inflammatory cascades, vascular integrity, and immune modulation within the intestinal tract. Finally, we will assess therapeutic interventions targeting PAF in NEC management and evaluate the efficacy and limitations of current therapeutic intervention strategies.

## Introduction and background

Necrotizing enterocolitis (NEC) is a life-threatening gastrointestinal disorder that primarily affects premature and very-low-birth-weight infants. Characterized by inflammation and necrosis of the intestinal wall, NEC typically presents within the first few weeks of life and remains one of the most serious complications in neonatal intensive care units (NICUs). The condition affects approximately 1-7% of NICU admissions, with the incidence rising to 10% in infants weighing less than 1,500 grams. Mortality rates can reach as high as 50% in severe cases, highlighting the urgent need for improved prevention and treatment strategies.

Premature infants are particularly vulnerable to NEC due to several developmental immaturities: reduced gastrointestinal motility, underdeveloped intestinal barriers, and impaired immune responses. These vulnerabilities make their intestines more susceptible to injury, microbial dysbiosis, and inflammation. A central mechanism in NEC pathogenesis involves exaggerated inflammatory signaling in the immature gut, especially through Toll-like receptors (TLRs), which are a class of proteins that detect microbial molecules and initiate innate immune responses. Toll-like receptor 4 (TLR4), in particular, is overexpressed in premature intestines and has been shown to mediate intestinal injury, inhibit epithelial repair, and exacerbate barrier breakdown in response to bacterial colonization.

Another key pathological factor is platelet-activating factor (PAF), a potent proinflammatory phospholipid produced by mast cells, monocytes, and macrophages. PAF contributes to NEC by promoting intestinal inflammation, apoptosis, vascular dysfunction, and hypoxia-related necrosis. Its effects are magnified in the context of reduced activity of PAF-acetylhydrolase, the enzyme responsible for its degradation, which is often deficient in preterm neonates. The combined interaction of PAF signaling and TLR4 activation creates a harmful inflammatory environment that compromises intestinal integrity and fuels NEC progression.

In addition to inflammatory mechanisms, NEC is associated with disruptions in the intestinal microbiome, altered epithelial differentiation, and compromised crypt development within the immature gut. Although NEC can occur in full-term infants, premature neonates exhibit significantly higher rates, severity, and mortality. Emerging evidence supports the protective roles of breast milk, amniotic fluid, and targeted biological therapies, such as growth factors and PAF-modulating agents. These insights offer promising directions for the prevention and management of this devastating pediatric condition.

## Review

PAF normal physiology and pharmacological indications 

**Figure 1:**
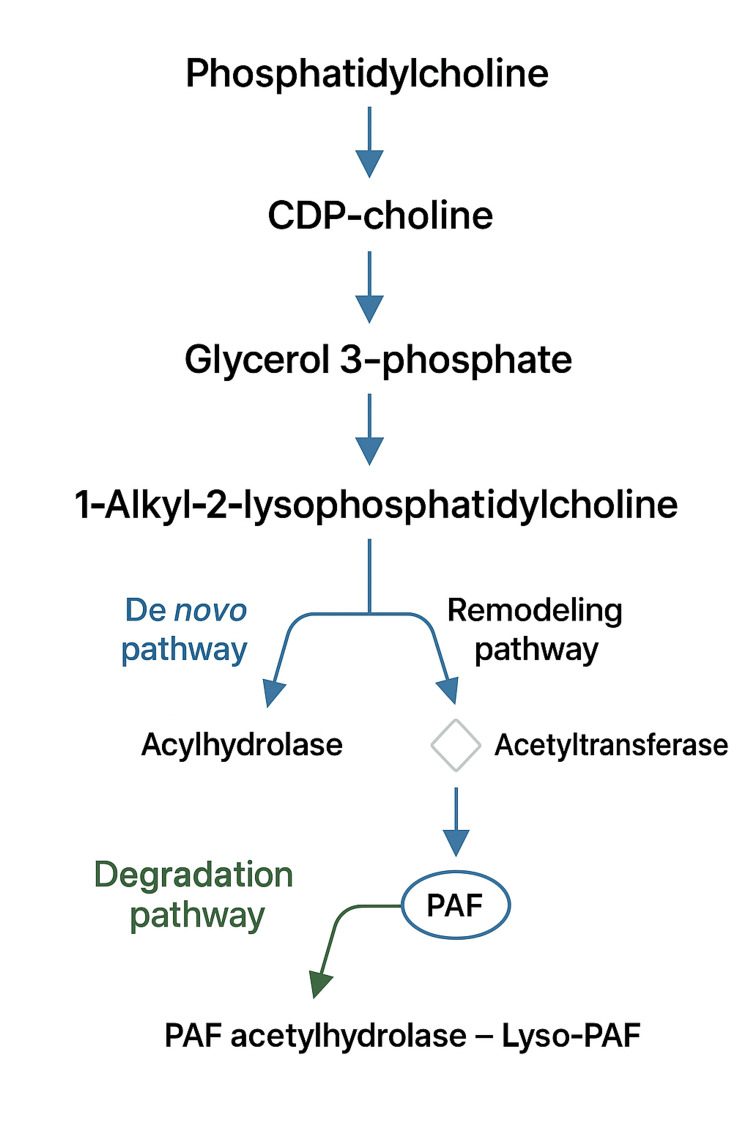
The metabolic pathway to synthesize platelet-activating factor, in the de novo and remodeling pathway, and the pathway to degrade platelet-activating factor. Created by the authors

PAF is a proinflammatory phospholipid that is involved in many different pathological and physiological processes, such as cellular functions; for example, it has a role in central nervous system signaling and inflammatory cascades [1-3]. It is also involved in apoptosis, physiological inflammation, wound healing, and angiogenesis. PAF is synthesized and secreted by mast cells, monocytes, and macrophages [4]. Figure 1 demonstrates the de novo and remodeling pathways of PAF synthesis [2,4,5]. The remodeling pathway is thought to be responsible for the production of PAF involved in inflammation, while the de novo pathway is thought to maintain basal levels of PAF in the body [2,6]. PAF acts via G protein activating phosphatidylinositol-specific phospholipase C by binding to a seven-transmembrane PAF receptor to trigger several downstream intracellular cascades for thrombotic and inflammatory events [1,4,5,7-9]. The regulation of PAF is dependent on which pathway is utilized. As seen in Figure 1, PAF acetyltransferase is the main regulatory enzyme in the remodeling pathway, while DTT-insensitive Cholinephosphotransferase is the main regulatory enzyme in the de novo pathway [2,4,6]. The plasma levels of PAF are low in healthy individuals; however, they are increased in many diseases such as cancer, renal diseases, and NEC. In pathological conditions, it has been observed that PAF has a role in cardiac dysfunction, such as cardiac anaphylaxis, hemorrhage, and septic shock [1,7,10,11].

It has been seen that PAF inhibitors can have clinical use in suppressing PAF-induced diseases and treating PAF-induced diseases [2,11]. PAF inhibitors are categorized into two different classifications, non-specific and specific, based on how they interact with the PAF receptor [2,12-14]. Non-specific inhibitors have a role in the PAF signal transduction pathway; however, due to their low specificity, they have limited pharmacological value. Specific inhibitors can bind either competitively or noncompetitively with the PAF receptor and hold potential therapeutic value.

NEC pathogenesis

Much of the pathogenesis related to NEC in premature infants centers around inflammatory mechanisms and dysregulated immune response in the premature intestine [15-19]. In addition, the development of NEC is primarily attributed to the breakdown of the intestinal barrier and disruption of microbiome integrity [19-22]. Current understanding of the progression of NEC focuses on an exaggerated immune response improperly balanced with reduced repair mechanisms [15,16]. Compared to adult intestinal mucosa, the intestinal epithelium of premature infants is predisposed to underdeveloped and immature intestinal epithelial cells, which are susceptible to bacterial invasion and NEC secondary to ischemia, inflammation, and injury [15,19]. Premature infants may also lack sufficient adaptive immune response from maternal antibodies. Factors such as gestational age, mode of delivery, feeding methods, and early antibiotic usage contribute to lower gut microbial diversity in preterm and very low birth weight infants, hindering the colonization of probiotic species like *Bacteroides*, *Bifidobacterium*, and *Atopobium* [21,22]. 

Risk factors influencing NEC development include low gestational age, low birth weight, chorioamnionitis, mechanical ventilation, genetic predisposition, intestinal immaturity, microvascular tone changes, and abnormal microbial colonization [23-25]. Genetics and immunological responses, particularly the upregulation of TLR-4, are implicated in NEC initiation [23,26,27]. Prematurity consistently emerges as a significant risk due to incomplete intestinal maturity, circulatory regulation, and immune barrier functions [24]. Immediate interventions like antibiotics, glucocorticoids, and delayed breastfeeding in premature neonates can impede normal microbiome development, heightening the risk of intestinal maldevelopment [23-25]. Because of sterility in the utero fetal environment, the initial exposure to bacteria during birth and colostrum consumption becomes essential to promoting infant immune system maturation [23-27]. The absence of gut flora creates a pro-inflammatory environment, leading to gut injury through immune dysregulation, decreased barrier maturation, and proliferation of other microorganisms [23-25,27].

TLR4 signaling and NEC

**Figure 2:**
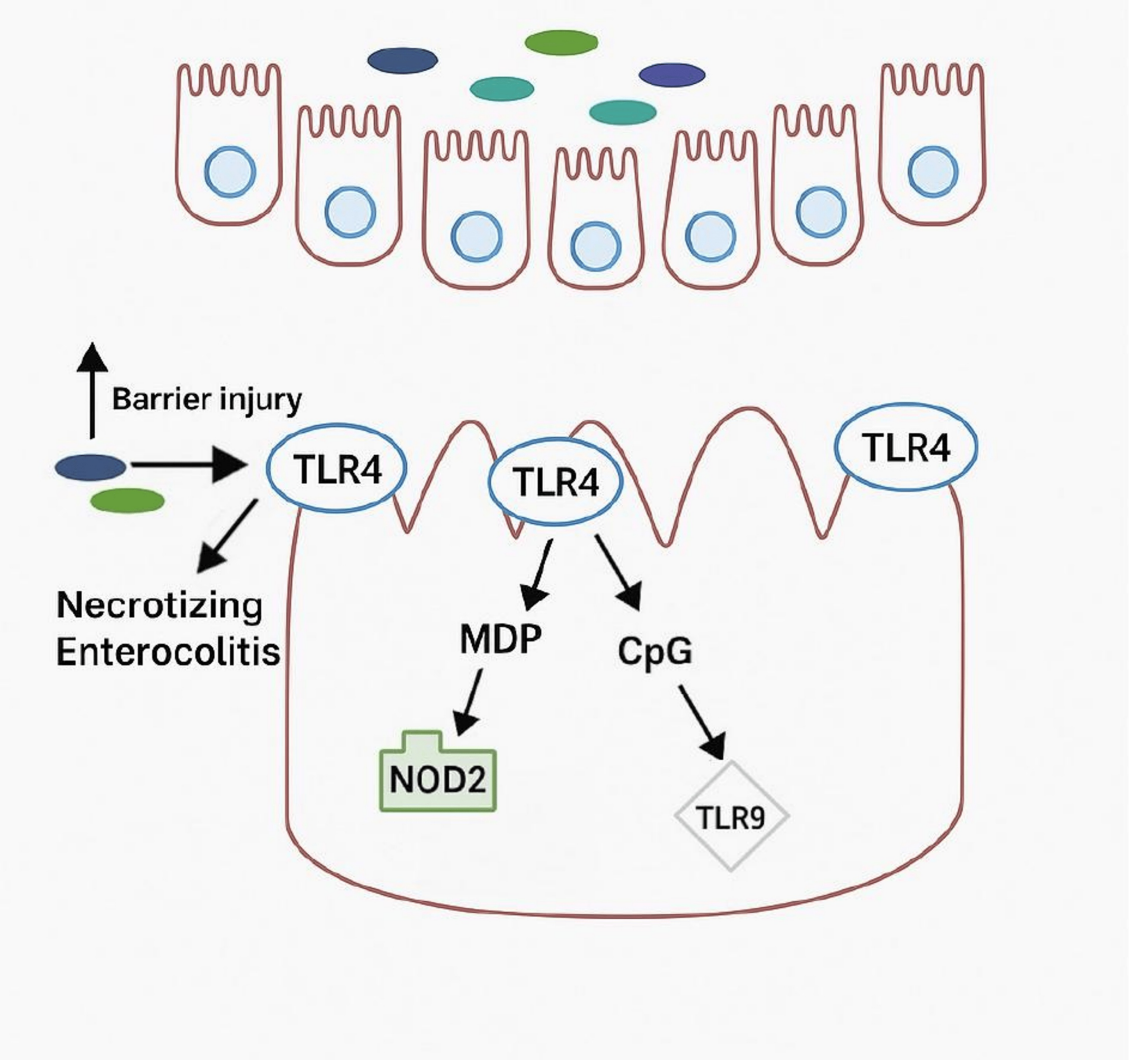
The pathway of necrotizing enterocolitis. In premature infants, the barrier in the gastrointestinal tract is damaged, allowing microflora to enter, activating TLR4, and causing necrotizing enterocolitis. TLR: toll-like receptor Created by the authors.

Known risk factors of NEC, such as prematurity and bacterial colonization, demonstrate how high levels of inflammation lead to ischemia [16,18,19]. As shown in Figure 2, TLR4, a toll-like receptor known to play a significant part in proinflammatory and innate immunity via recognition of lipopolysaccharide (LPS) on gram-negative bacteria, regulates normal intestinal epithelial growth [16,18]. The LPS ligand of TLR4 inhibits enterocyte migration and reduces enterocyte proliferation through beta-catenin signaling inhibition [20,22]. Excessive inflammation disrupts the gut microbiome, predisposing premature neonates to NEC [21,22]. Although microorganisms from the phyla *Firmicutes*, *Proteobacteria*, and *Clostridium* spp. are implicated in NEC, there is no known association with a particular microorganism [20]. Abnormal and elevated TLR4 signaling was expressed in intestinal samples from infants with NEC and in mice with NEC [16,28,29]. Mice that did not express TLR4 were consequently unable to develop NEC [16,28]. Moreover, the use of TLR4 inhibitors reduced the proinflammatory response in NEC-positive human tissue and successfully treated NEC in mouse models.

The activation of TLR4 in the immature intestinal epithelium is vital to NEC progression. TLR4 expression by intestinal epithelial cells maintains tight junctions crucial to gut barrier protection. It also activates nuclear factor-kB (NF-kB), leading to a subsequent acute inflammatory response [16,28,20]. Due to underdeveloped intestinal epithelial cells, subsequent overactivation of TLR4, and increased gut permeability, pathogenic bacteria translocate through the mesentery and interact with TLR4 [16,30]. This causes intestinal ischemia within the blood vessels. PAF has also been shown to play a role in inducing over-expressed levels of TLR4 and will be discussed in later sections [29,30].

Intestinal defense against NEC

**Figure 3:**
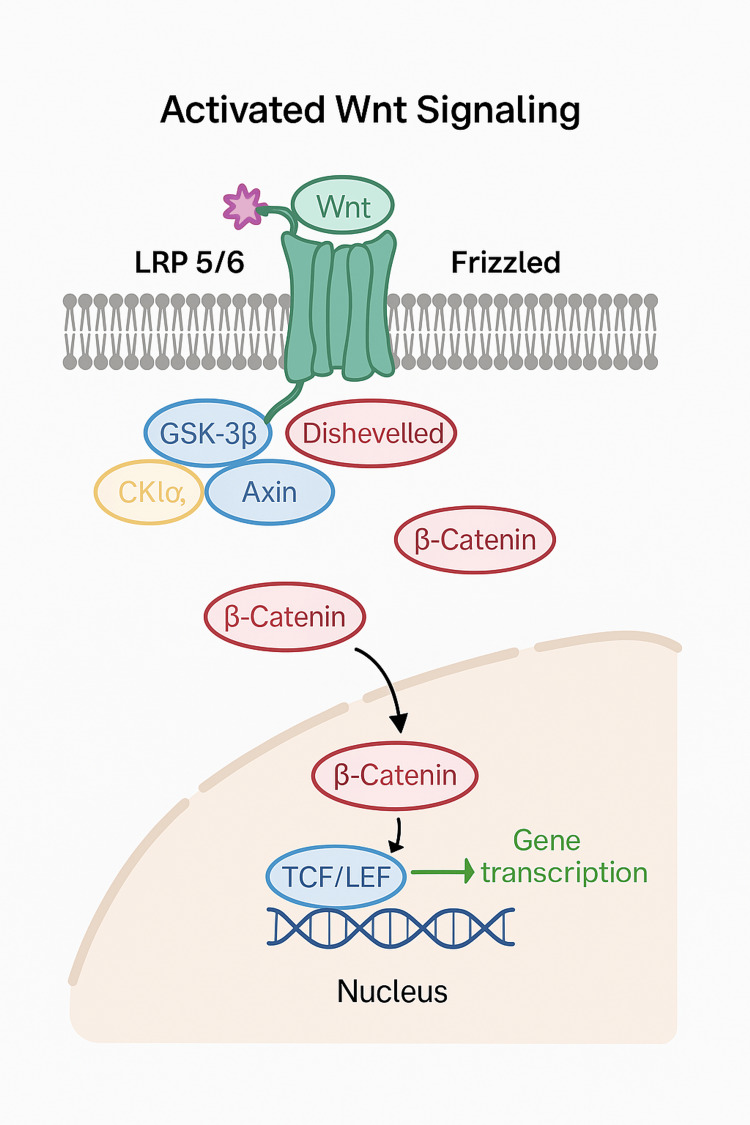
Activated WNT signaling pathways. Created by the authors.

**Figure 4:**
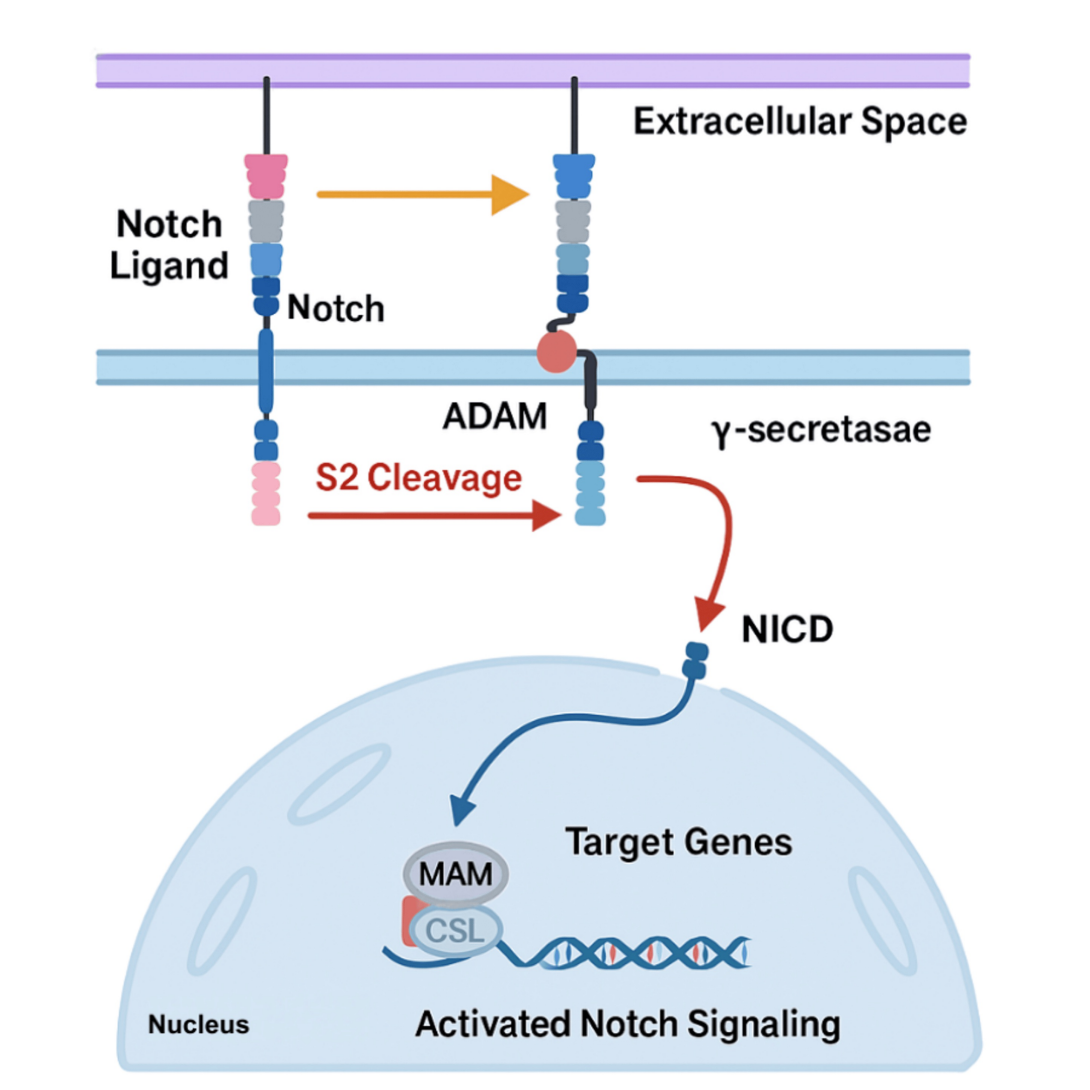
Activated notch signaling pathways. Created by the authors.

The small intestine is a sophisticated, multilayered organ with diverse secretory, neuroendocrine, and regulatory components. Within the intestine, the crypts of Lieberkuhn house both stem cells and Paneth cells. Paneth cells crucially guide the differentiation of columnar cells [31,32]. Stem cells within the crypts give rise to three essential cell types: enterocytes, responsible for both nutrient digestion and absorption, goblet cells that release mucin for a protective mucosal barrier, and enteroendocrine cells involved in energy balance sensing [23-24,26,33]. As shown in Figure 3 and Figure 4, the WNT and NOTCH signaling regulate cellular differentiation and are dispersed in concentration gradient patterns. WNT signaling is more pronounced in Paneth cells [32,33].

NEC manifests as deficiencies in GI motility and mucosal membrane integrity, which subsequently reduces GI secretions and promotes bacterial overgrowth. Delayed intestinal epithelial development contributes to decreased pathogen and toxin removal, fostering inflammation [23,24]. Damage to Paneth cells, goblet cells, and intestinal stem cells increases NEC risk as well [23,24,26,33]. Insufficient mucin production by goblet cells, attributed to increases in the NOTCH pathway, may allow pathogens and toxins to escape into the systemic circulation [24,26]. Suppression of the NOTCH pathway correlates with decreased NEC severity and increased goblet cell formation.

The mucous membrane layer, crucial for protection against bacteria and toxins, is less securely attached to the lumen in the small intestine compared to the colon, rendering it more susceptible to bacterial injury [20-22]. Premature neonates, due to the incomplete formation of their enteric nervous system, face a higher risk of bacterial extravasation through the membrane, increasing the likelihood of NEC development [20,21]. Damage to tight junctions leads to reduced proliferation and differentiation of stem cells, along with increased apoptotic rates [20,22,34].

Paneth cells are essential for defense against bacterial growth and produce antimicrobial materials such as alpha-defensins, cathelicidins, lysosomes, and phospholipase A2 [20,21]. NEC develops in the neonatal period due to decreased production of these antimicrobial peptides and reduced differentiation of intestinal cells [20,34]. Intestinal cell regeneration, governed by the Wnt/B-catenin pathway, shown in Figure 3, is crucial for minimizing NEC injury [20,35], as NEC is associated with a reduction in WNT/B-catenin signaling in humans. Inhibition of different parts of the signaling pathway yields varied effects in NEC, such as decreased enterocyte proliferation and impaired intestinal stem cell activity [20,34,35]. IL-22, linked to an inflammation-dampening pathway, triggers intestinal epithelial cell regeneration [36]. Inhibition of this signaling pathway increases intestinal damage and necrosis, which is exacerbated by lower IL-22 concentration in the neonatal period [30,36]. Myeloid differentiation protein 2 (MD2) is upregulated in the LPS/TLR4 signaling pathway in NEC patients and activates downstream effectors like NF-κB, a key player in the proinflammatory response [34-36]. MD2 is a glycoprotein that is involved in the LPS recognition by toll-like receptors. Upon LPS binding to MD2, it forms a complex with TLR4, promoting dimerization [34]. Subsequently, myeloid differentiation factor 88 (MyD88) is recruited, initiating downstream signaling pathways, notably the nuclear factor kappa-B (NF-κB) pathway [34,37,38]. An overexpression of MD2 leads to the downregulation of mucins and tight junction proteins in NEC [20,34].

In normal anatomy, blood flow in the intestine is greatest at the base of the villi and lowest at the distal ends. In hypoxic conditions, the distal ends are primarily damaged, creating fluctuations in cellular consistency and heightened gut inflammation [22-25]. The interaction between TLR4 and bacteria in the intestinal lumen and blood vessels impairs blood flow regulation, causing an imbalance between endothelin-1 and nitric oxide. This reduces endothelial NO synthase expression, diminishes NO production, and increases endothelin-1-induced vasoconstriction [22,23]. Hypoxia increases oxidative stress and triggers an inflammatory response. PAF, generated by platelets and inflammatory mediators in response to ischemic conditions, can escalate intestinal injury to necrosis [22-24].

NEC in premature infants vs. full-term infants

Premature Infants and NEC

While NEC is a complex and multifactorial gastrointestinal condition that affects both premature and full-term infants, the clinical presentation and severity of the condition differ between the two [27,37-40]. Premature infants, particularly those born before or between 28 and 32 weeks of gestation, are at increased risk for developing NEC [40-42]. These infants typically have a low birth weight and exhibit lower microbial diversity, leaving their immature gastrointestinal tract susceptible to inflammation, injury, and diseases such as NEC [29,38,40,43]. One contributing factor is the delayed maturation of the intestinal tract [38,40,44]. The microbial dysbiosis in the gut could also contribute to a late onset of NEC [40,43,44]. For example, a premature infant born at 27 weeks may exhibit NEC symptoms within three to five weeks of their birth, considered late-onset [18,42]. As previously mentioned, factors such as cesarean delivery, formula-feeding, and early antibiotic use, common in NICU settings, have been associated with reduced microbial diversity, whereas breastfeeding tends to promote a more beneficial and stable gut microbiota. [18,27,38,41,43]. Studies have shown the significance of the vaginal flora as the first source of microbial colonization and can maintain intestinal homeostasis and provide protection from intestinal injury [27,38]. An infant born vaginally tends to have a gut microbiota more closely resembling their mother’s microbiota than one delivered via cesarean section, which tends to resemble the microbiota of the mother’s skin [38,44]. Preterm infants also tend to have a low birth weight and tend to have a decreased ability to breastfeed, thus decreasing the commensal bacteria that come from the mother [38,41]. In addition, the incidence of NEC is lower when infants are fed breast milk as opposed to formula [38,43,45].

The consequences are more severe for preterm infants. If NEC continues to progress, it can often lead to complications such as perforation of the intestines, which requires a bowel resection, or neurodevelopmental delays [17,26,37,38]. The human microbiota contributes to proper brain development, and any dysfunction or dysregulation of it can lead to altered expressions of insulin-like growth factor 1, which can be associated with altered perinatal growth and abnormal neurodevelopment [17,37,39]. 

Full-Term Infants and NEC

Although NEC is not as prevalent for full-term infants, it is still a serious and potentially life-threatening condition. Full-term infants are more likely to have a lower stage of disease and better prognosis compared to those born prematurely [18,46]. In addition, there is an inverse relationship between the gestational age and onset of postnatal symptoms [18,44,46]. The more premature an infant is born, the later the onset of symptoms, whereas full-term infants (considered ≥37 weeks) can exhibit symptoms within the first two weeks of birth [18,40,41,46]. One study compared outcomes and comorbidities between premature and full-term infants who were in the NICU and had a NEC diagnosis [46,47]. The study found a significant difference in an increased amount of full-term infants who had underlying congenital cardiac anomalies compared to premature infants.

NEC and the role of PAF in premature infants vs. full-term infants

NEC can develop in both term and preterm infants, but preterm infants afflicted with NEC exhibit a significantly higher frequency, severity, and mortality than term infants [48-50]. In fact, roughly 5% of all low-birth-weight babies (<1,500 g at birth) are afflicted with NEC, as are around 10% of all extremely low-birth-weight babies (<1,000 g at birth) [48,51,52]. The increased risk and more severe progression of NEC in preterm infants is attributed to the relationship between PAF and the enzyme responsible for its degradation, PAF-acetylhydrolase (PAF-AH) [52-54].

As stated, PAF is a proinflammatory mediator that, when in excess, plays a key role in the development and progression of NEC [55-58]. To limit PAF-induced inflammation to appropriate levels, PAF is catabolized by PAF-AH [52-54]. The relationship between PAF, PAF-AH, and NEC has been well documented; studies with animal models have shown administration of PAF to favor the development of NEC, while PAF-AH has been shown to protect against NEC [56,59,60,61]. 

Premature babies are born with high circulating levels of PAF and low levels of PAF-AH [60,62]. This imbalance disrupts the regulatory actions of PAF-AH on PAF, leaving the infant more likely to develop NEC [60-62]. Of note, parenteral feedings of preterm infants have been shown to promote the production of PAF, but it remains unclear if this increased PAF production definitively leads to NEC development [63-65].

In addition to promoting the inflammation that ultimately causes NEC, it has been suggested that PAF may play an additional role in NEC development. As discussed, prematurity and low birth weight are important risk factors for NEC [48,49,52,53,54]. Increased levels of PAF have been implicated as a cause of preterm delivery due to its contribution to the production of PGE2 and the subsequent stimulation of myometrial contraction [65-67]. Given the increased risk of NEC development with low birth weight, PAF may also support the pathogenesis of NEC by putting the infant at a greater risk of being born prematurely. 

Role of PAF in intestinal injury 

Intestinal injury leading to NEC is attributed to decreased blood perfusion, microbial colonization, or compromised immunological defenses [22,39,68,69]. The specific mechanisms by which this intestinal injury occurs are yet to be comprehensively understood. One proposition is the role of increased PAF in the pathogenesis of intestinal damage [70-72]. The role of hypoxia in decreased blood perfusion and the development of necrosis within the intestines is well established [72-75]. However, the exact mechanism of hypoxia remains unclear, and PAF may likely contribute [9,76]. Caplan et al. observed the manner in which rats receiving PAF receptor antagonists display early signs of intestinal necrosis yet preserve normal intestinal perfusion, which then halts further development of necrosis [77-79]. Because PAF receptor antagonists prevent eventual bowel injury, these results indicate the detrimental yet central role of PAF in the pathogenesis of intestinal necrosis via hypoxia [71,77,80]. Intestinal perfusion is regulated, in part, by neuronal nitric oxide synthase, and its stabilization by tetrahydrobiopterin prevents PAF from inducing intestinal injury [81-83]. Furthermore, decreased nitric oxide pathway activity in premature infants precedes NEC diagnosis in piglets [17]. PAF induces intestinal mucosal barrier permeability through tyrosine phosphorylation of E-cadherin and cytoskeletal alteration of enterocytes [83-85]. Hypoperfusion and hyperpermeability of enterocytes, including that induced by PAF, likely contribute to overall intestinal injury, including that of NEC [86-88]. 

Compromised immunological defenses are another proposed mechanism of PAF-mediated injury of intestinal cells [22,39,68,69]. Apoptosis is one specific method understood to involve PAF. By activating Ca2+-dependent Cl− channels ClC-3, PAF induces intracellular intestinal epithelial cell acidosis, leading to eventual apoptosis via Caspase 3 activation and DNA fragmentation [89,90]. Further evidence supports this role, as Bcl-2 (anti-apoptotic) activity halts PAF-induced mitochondrial BAX translocation and therefore caspase activation [91,92]. Its influence on BAX translocation, caspase activation, and DNA fragmentation, as well as its inhibition by Bcl-2, provides additional support for the role of PAF in intestinal cell damage via apoptosis [92-94].

Treatment

Mainstay treatments of NEC include enteral feedings, gastric decompression, and antibiotics. Indications for surgery are individualized, although absolute indications for surgery exist, such as perforations [95,96,97]. Syed et al. noted that surgery was only more effective if there was no perforation. Earlier occurrence and diagnosis of NEC, younger age, NSAID usage, and the absence of enteral feeding before the onset of NEC correlate with higher surgical need in infants [97-99]. However, human breast milk remains one therapy that provides the most benefit in patients with NEC [100,101]. When compared to exclusively formula-fed counterparts, children fed with breast milk were six to 10 times less likely to develop NEC [102]. A threshold as low as 50% human breast milk feedings can benefit patients [103]. One systematic review reported that donor milk does not seem to provide a clear benefit for reducing the risk of NEC [104]. However, exclusive human breast milk is protective, and a mother’s breast milk remains of the highest benefit [103,104]. 

As stated previously, breast milk contains nitrous oxide (NO), oligosaccharides, cytokines, and chemokines. Decreased nitrous oxide can contribute to ischemic necrosis and cellular death due to its role in regulating blood flow. Prior research has established that supplementation of breast milk is protective. When considering the correlation of increased NO in breast milk and the benefits of breast milk, this could explain why breast milk is beneficial.

Breast milk also houses IgA, an immunoglobulin essential for mucosal defense and maintenance of gut barrier integrity. There is 55% more IgA in maternal breast milk (MBM) than donor breast milk, and a 49% higher concentration of IgA in infants supplemented with MBM when compared against infants given donor milk [17]. Rat pups given milk from IgA-deficient mothers are at increased risk of intestinal infections and a decrease in commensal bacteria [105,106]. Antibiotics may also decrease commensal bacteria. These findings may indicate not only why breast milk remains one of the best therapeutic options but also why other treatments are not as successful and could increase the risk of NEC diagnosis. 

TLR4 is directly implicated in the development of NEC [30,102] by enhancing PAF release from neutrophils. It is thought that breast milk counteracts TLR-4-mediated disruption of the Wnt and Notch pathways, seen in Figure 3, both of which are responsible for maturation of intestinal cells [30,107]. Human milk rescues both LPS- and PAF-induced intestinal cell death in vitro by promoting cellular proliferation and inhibiting inflammatory cellular pathways [108]. It is thought that antibiotics decrease TLR4 signaling as well, which would also protect against NEC [109]. 

A recent wave of studies indicates that administering amniotic fluid (AF) to infants has a protective effect against NEC [104,110,111]. Formula supplemented with amniotic fluid diminished NEC injury in rat pups [106,111]. Similar to breast milk, AF has many protective components, such as antimicrobial, growth, and trophic factors [104,105]. We have noted previously that preterm infants have an increased risk of NEC because of lesser GI development. Swallowing amniotic fluid in utero can induce epithelial regeneration and renewal, which is essential for gastrointestinal development [100,104]. 

Growth factors TGFb, epidermal growth factor (EGF), IGF, and oligosaccharides show promise in treating NEC by reducing inflammation [88,101,111-114]. Good et al. found EGF in amniotic fluid decreased LPS signaling in fetal intestines by inhibiting TLR-4 [88]. A correlated knockout of EGF in human milk has been associated with an increase in cellular death in vitro [108]. These findings imply both human milk and amniotic fluid may act protectively through the EGF pathway, although more data are needed to validate these results. Administration of cells through extracellular vesicles reduces intestinal injury and increases cellular proliferation [115-117]. Other recent works demonstrate that both stem cells and extracellular vesicles activate the Wnt pathway, which then reduces inflammation and induces maturation and proliferation of intestinal epithelial cells [118]. When used in vitro, colostrum-derived exosomes reduced NEC score [105,113]. Furthermore, various studies have found that both amniotic fluid stem cells-derived exosomes and breast milk-derived exosomes reduced NEC-associated injury [108,118,119]. 

Exogenous stem cells reduce intestinal injury and increase cellular proliferation [115-117]. Breast milk is also a source of stem cells. It is thought that breast milk may regenerate cells and repair cellular injury, although more studies are needed to elucidate this effect [100,107,119]. Administration of mesenchymal stem cells in rats rescues NEC-mediated injury and produces similar beneficial effects [110,112,116,120]. Amniotic stem cells (AFSCs) are another therapy for NEC [101,104,121-123]. AFSCs show potential as a preventative therapy, potentially because they preserve epithelial tight junctions and lessen permeability of intestinal cells [60,103,105,119,121,122]. 

Increase in PAF and decrease of the protective PAH-AH have been correlated to the development of NEC [123-125]. Pretreatment with dexamethasone and medroxyprogesterone subsequently increased PAF-AH and prevented intestinal damage in rats [60,106,125]. In addition, the supplementation of enteral PAF-AH significantly reduced NEC when compared to physiologically normal controls [124-126]. PAF-AH is present in breast milk as well, perhaps yet another explanation as to its therapeutic benefits [124]. 

There is no uniform antibiotic treatment to treat NEC [127-131], and up to 20 different combinations of antibiotics have been used within the past eight years [131-134]. Warner et al found that broad-spectrum antibiotics most likely do not reduce mortality in infants with NEC [134]. Furthermore, there was no reduction in mortality when anaerobic antimicrobial therapy was used in patients with stage 3 NEC [134,135]. Other studies have shown no difference in mortality when any combination of antibiotics is used [98,99,129]. However, prolonged use of antibiotics is widely considered detrimental, and current studies indicate empiric antibiotic usage for more than four days can increase the risk of NEC. More than 10 days of prophylactic antibiotics also increases risk [98,100,131]. It is thought that prolonged therapy reduces bacterial diversity in the intestine, which is associated with increased incidence of NEC [135,136]. 

Previously, there was agreement that prophylactic antibiotics can prevent NEC, but many of these studies are outdated. One recent group observed that empiric antibiotics given for less than 3 days are associated with a lower risk of NEC when compared to both long-term administration or no antibiotic treatment [89]. Prophylactic antibiotics, given to preterm pigs enterally, demonstrated protective effects against NEC [99,131,135]. In infants, other studies concluded that short-term antibiotic treatment given to preterm VLBW patients shortly after birth decreased the incidence of NEC when compared to counterparts who did not receive treatment [128-131]. Of note, bacterial load in the stool of infants with NEC was not significantly different from their healthy counterparts [136,137]. More studies are needed to conclusively determine their importance, as antibiotic treatment may not be necessary, and, as stated previously, may not lower mortality in infants. 

In the future, exosome-encapsulated nano therapies in various stem cells are expected to show promise [113,119,120]. Recent trends show efforts to characterize NEC with plasma protein marker profiles [116,117]. Other potential modalities include the introduction of probiotics, of which some meta-analyses have been performed [119,137]. If successful, early intervention, preemptive treatment, and diagnosis of NEC will likely improve mortality statistics in patients with NEC. However, more studies are needed to further examine these potential tools.

## Conclusions

NEC is a complex condition that has an intricate interplay of molecular, immunological, and physiological factors contributing to the pathogenesis of this gastrointestinal disease that primarily affects premature infants. The mechanisms associated with NEC range from dysregulated immune responses and compromised intestinal barriers to microbial dysbiosis. PAF has a pivotal role in the progression of NEC. Premature infants, marked by underdeveloped intestinal systems, are especially prone to NEC, with TLR4 signaling and PAF identified as central elements in its initiation and development. The interconnection between PAF and TLR4 presents a potential avenue for targeted therapeutic interventions to reduce the risk of NEC. There are many different treatment strategies available, including enteral feedings, antibiotics, and surgical interventions. It has been seen that breast milk has a protective effect and is a crucial therapeutic option as it provides a rich source of elements such as nitrous oxide, IgA, and growth factors. In addition, promising avenues involve the administration of amniotic fluid, extracellular vesicles, and stem cells, showcasing potential benefits in reducing inflammation and fostering cellular regeneration. These treatments are aimed at regulating PAF levels and its regulatory enzymes, such as PAF-AH, in premature infants who display PAF level imbalances. Overall, this review provides a comprehensive overview of NEC, bridging the gap between molecular intricacies and clinical implications. The collective understanding of NEC's pathophysiology and potential interventions opens avenues for further research, aiming to refine strategies for early diagnosis, targeted treatment, and improved outcomes in infants at risk of this serious gastrointestinal condition.

## References

[1] Snyder F (1995). Platelet-Activating Factor and its Analogs: Metabolic Pathways and Related Intracellular Processes. BBA - Mol Cell Biol Lipids.

[2] Lordan R, Tsoupras A, Zabetakis I, Demopoulos CA (2019). Forty years since the structural elucidation of platelet-activating factor (PAF): historical, current, and future research perspectives. Molecules.

[3] Chao W, Olson MS (1993). Platelet-activating factor: receptors and signal transduction. Biochem J.

[4] Andrades E, Clarós M, Torres JV (2022). New transcriptome and clinical findings of platelet-activating factor in chronic spontaneous urticaria: pathogenic and treatment relevance. Biofactors.

[5] Muñoz-Cano RM, Casas-Saucedo R, Valero Santiago A, Bobolea I, Ribó P, Mullol J (2019). Platelet-activating factor (PAF) in allergic rhinitis: clinical and therapeutic implications. J Clin Med.

[6] Detopoulou P, Nomikos T, Fragopoulou E, Stamatakis G, Panagiotakos DB, Antonopoulou S (2012). PAF and its metabolic enzymes in healthy volunteers: interrelations and correlations with basic characteristics. Prostaglandins Other Lipid Mediat.

[7] Montrucchio G, Alloatti G, Camussi G (2000). Role of platelet-activating factor in cardiovascular pathophysiology. Physiol Rev.

[8] Deng M, Guo H, Tam JW (2019). Platelet-activating factor (PAF) mediates NLRP3-NEK7 inflammasome induction independently of PAFR. J Exp Med.

[9] Deng Y, Fang W, Li Y (2009). Blood-brain barrier breakdown by PAF and protection by XQ-1H due to antagonism of PAF effects. Eur J Pharmacol.

[10] Sharif NA (2022). PAF-induced inflammatory and immuno-allergic ophthalmic diseases and their mitigation with PAF receptor antagonists: cell and nuclear effects. Biofactors.

[11] Travers JB, Rohan JG, Sahu RP (2021). New insights into the pathologic roles of the platelet-activating factor system. Front Endocrinol (Lausanne).

[12] Demopoulos CA, Karantonis HC, Antonopoulou S (2003). Platelet activating factor — a molecular link between atherosclerosis theories. Eur J Lipid Sci Technol.

[13] Papakonstantinou VD, Lagopati N, Tsilibary EC, Demopoulos CA, Philippopoulos AI (2017). A review on platelet activating factor inhibitors: could a new class of potent metal-based anti-inflammatory drugs induce anticancer properties?. Bioinorg Chem Appl.

[14] Honda Z, Ishii S, Shimizu T (2002). Platelet-activating factor receptor. J Biochem.

[15] Cai X, Golubkova A, Hunter CJ (2022). Advances in our understanding of the molecular pathogenesis of necrotizing enterocolitis. BMC Pediatr.

[16] Hackam DJ, Sodhi CP (2018). Toll-like receptor-mediated intestinal inflammatory imbalance in the pathogenesis of necrotizing enterocolitis. Cell Mol Gastroenterol Hepatol.

[17] Hirai C, Ichiba H, Saito M, Shintaku H, Yamano T, Kusuda S (2002). Trophic effect of multiple growth factors in amniotic fluid or human milk on cultured human fetal small intestinal cells. J Pediatr Gastroenterol Nutr.

[18] Niño DF, Sodhi CP, Hackam DJ (2016). Necrotizing enterocolitis: new insights into pathogenesis and mechanisms. Nat Rev Gastroenterol Hepatol.

[19] Tanner SM, Berryhill TF, Ellenburg JL, Jilling T, Cleveland DS, Lorenz RG, Martin CA (2015). Pathogenesis of necrotizing enterocolitis: modeling the innate immune response. Am J Pathol.

[20] Managlia E, Yan X, De Plaen IG (2022). Intestinal epithelial barrier function and necrotizing enterocolitis. Newborn (Clarksville).

[21] Lee JK, Hern Tan LT, Ramadas A, Ab Mutalib NS, Lee LH (2020). Exploring the role of gut bacteria in health and disease in preterm neonates. Int J Environ Res Public Health.

[22] Kaplina A, Kononova S, Zaikova E, Pervunina T, Petrova N, Sitkin S (2023). Necrotizing enterocolitis: the role of hypoxia, gut microbiome, and microbial metabolites. Int J Mol Sci.

[23] Meister AL, Doheny KK, Travagli RA (2020). Necrotizing enterocolitis: it's not all in the gut. Exp Biol Med (Maywood).

[24] Schnabl KL, Van Aerde JE, Thomson AB, Clandinin MT (2008). Necrotizing enterocolitis: a multifactorial disease with no cure. World J Gastroenterol.

[25] Alganabi M, Lee C, Bindi E, Li B, Pierro A (2019). Recent advances in understanding necrotizing enterocolitis. F1000Res.

[26] Venkatraman A, Yu W, Nitkin C, Sampath V (2021). Intestinal stem cell development in the neonatal gut: pathways regulating development and relevance to necrotizing enterocolitis. Cells.

[27] Niemarkt HJ, De Meij TG, van Ganzewinkel CJ, de Boer NK, Andriessen P, Hütten MC, Kramer BW (2019). Necrotizing enterocolitis, gut microbiota, and brain development: role of the brain-gut axis. Neonatology.

[28] Kovler ML, Gonzalez Salazar AJ, Fulton WB (2021). Toll-like receptor 4-mediated enteric glia loss is critical for the development of necrotizing enterocolitis. Sci Transl Med.

[29] Robertson SA, Hutchinson MR, Rice KC (2020). Targeting Toll-like receptor-4 to tackle preterm birth and fetal inflammatory injury. Clin Transl Immunology.

[30] Mihi B, Good M (2019). Impact of toll-like receptor 4 signaling in necrotizing enterocolitis: the state of the science. Clin Perinatol.

[31] Goto Y, Obata T, Kunisawa J (2014). Innate lymphoid cells regulate intestinal epithelial cell glycosylation. Science.

[32] Lueschow SR, McElroy SJ (2020). The paneth cell: the curator and defender of the immature small intestine. Front Immunol.

[33] Gassler N (2017). Paneth cells in intestinal physiology and pathophysiology. World J Gastrointest Pathophysiol.

[34] Huang D, Wang P, Chen J, Li Y, Zhu M, Tang Y, Zhou W (2022). Selective targeting of MD2 attenuates intestinal inflammation and prevents neonatal necrotizing enterocolitis by suppressing TLR4 signaling. Front Immunol.

[35] Li B, Lee C, Cadete M (2019). Impaired Wnt/β-catenin pathway leads to dysfunction of intestinal regeneration during necrotizing enterocolitis. Cell Death Dis.

[36] Mihi B, Gong Q, Nolan LS (2021). Interleukin-22 signaling attenuates necrotizing enterocolitis by promoting epithelial cell regeneration. Cell Rep Med.

[37] Yang Y, Sheng Y, Wang J (2021). Aureusidin derivative CNQX inhibits chronic colitis inflammation and mucosal barrier damage by targeting myeloid differentiation 2 protein. J Cell Mol Med.

[38] Baranowski JR, Claud EC (2019). Necrotizing enterocolitis and the preterm infant microbiome. Adv Exp Med Biol.

[39] Neu J, Walker WA (2011). Necrotizing enterocolitis. N Engl J Med.

[40] Pammi M, Cope J, Tarr PI (2017). Intestinal dysbiosis in preterm infants preceding necrotizing enterocolitis: a systematic review and meta-analysis. Microbiome.

[41] Kinstlinger N, Fink A, Gordon S (2021). Is necrotizing enterocolitis the same disease in term and preterm infants?. J Pediatr Surg.

[42] Nair J, Longendyke R, Lakshminrusimha S (2018). Necrotizing enterocolitis in moderate preterm infants. Biomed Res Int.

[43] Elgin TG, Kern SL, McElroy SJ (2016). Development of the neonatal intestinal microbiome and its association with necrotizing enterocolitis. Clin Ther.

[44] Denning NL, Prince JM (2018). Neonatal intestinal dysbiosis in necrotizing enterocolitis. Mol Med.

[45] Velazco CS, Fullerton BS, Hong CR (2017). Morbidity and mortality among "big" babies who develop necrotizing enterocolitis: a prospective multicenter cohort analysis. J Pediatr Surg.

[46] Overman RE Jr, Criss CN, Gadepalli SK (2019). Necrotizing enterocolitis in term neonates: a different disease process?. J Pediatr Surg.

[47] Li QY, An Y, Liu L, Wang XQ, Chen S, Wang ZL, Li LQ (2017). Differences in the clinical characteristics of early- and late-onset necrotizing enterocolitis in full-term infants: a retrospective case-control study. Sci Rep.

[48] Amer MD, Hedlund E, Rochester J, Caplan MS (2004). Platelet-activating factor concentration in the stool of human newborns: effects of enteral feeding and neonatal necrotizing enterocolitis. Biol Neonate.

[49] Frid G, Reppucci M, Lum T, Paul M, Seiden H, Coakley BA (2021). Comparison of necrotizing enterocolitis in pre-mature infants vs. term-born infants with congenital heart disease. Front Pediatr.

[50] Gregory KE, Deforge CE, Natale KM, Phillips M, Van Marter LJ (2011). Necrotizing enterocolitis in the premature infant: neonatal nursing assessment, disease pathogenesis, and clinical presentation. Adv Neonatal Care.

[51] Hunter CJ, Upperman JS, Ford HR, Camerini V (2008). Understanding the susceptibility of the premature infant to necrotizing enterocolitis (NEC). Pediatr Res.

[52] Patel BK, Shah JS (2012). Necrotizing enterocolitis in very low birth weight infants: a systemic review. ISRN Gastroenterol.

[53] Karasawa K, Harada A, Satoh N (2003). Plasma platelet activating factor-acetylhydrolase (PAF-AH). Prog Lipid Res.

[54] McIntyre TM, Prescott SM, Stafforini DM (2009). The emerging roles of PAF acetylhydrolase. J Lipid Res.

[55] Piwowarek KŁ, Rzeszotarska A, Korsak JŁ, Juszkiewicz A, Chciałowski A, Kruszewski J (2021). Clinical significance of plasma PAF acetylhydrolase activity measurements as a biomarker of anaphylaxis: cross-sectional study. PLoS One.

[56] Caplan MS, Lickerman M, Adler L, Dietsch GN, Yu A (1997). The role of recombinant platelet-activating factor acetylhydrolase in a neonatal rat model of necrotizing enterocolitis. Pediatr Res.

[57] Caplan MS, Simon D, Jilling T (2005). The role of PAF, TLR, and the inflammatory response in neonatal necrotizing enterocolitis. Semin Pediatr Surg.

[58] Caplan MS, Sun XM, Hsueh W (1991). Hypoxia, PAF, and necrotizing enterocolitis. Lipids.

[59] Upton JE, Grunebaum E, Sussman G, Vadas P (2022). Platelet activating factor (PAF): a mediator of inflammation. Biofactors.

[60] Furukawa M, Lee EL, Johnston JM (1993). Platelet-activating factor-induced ischemic bowel necrosis: the effect of platelet-activating factor acetylhydrolase. Pediatr Res.

[61] Lu J, Pierce M, Franklin A (2010). Dual roles of endogenous platelet-activating factor acetylhydrolase in a Murine model of necrotizing enterocolitis. Pediatric Research.

[62] Muguruma K, Gray PW, Tjoelker LW, Johnston JM (1997). The central role of PAF in necrotizing enterocolitis development. Adv Exp Med Biol.

[63] Cuna A, George L, Sampath V (2018). Genetic predisposition to necrotizing enterocolitis in premature infants: current knowledge, challenges, and future directions. Semin Fetal Neonatal Med.

[64] MacKendrick W, Hill N, Hsueh W, Caplan M (1993). Increase in plasma platelet-activating factor levels in enterally fed preterm infants. Biol Neonate.

[65] Moya FR, Eguchi H, Zhao B (1994). Platelet-activating factor acetylhydrolase in term and preterm human milk: a preliminary report. J Pediatr Gastroenterol Nutr.

[66] Hoffman DR, Romero R, Johnston JM (1990). Detection of platelet-activating factor in amniotic fluid of complicated pregnancies. J Pediatr Surg.

[67] Silver R, Caplan M, Kelly A (1992). Amniotic fluid platelet-activating factor (PAF) is elevated in patients with tocolytic failure and preterm delivery. Prostaglandins.

[68] Duess JW, Sampah ME, Lopez CM, Tsuboi K, Scheese DJ, Sodhi CP, Hackam DJ (2023). Necrotizing enterocolitis, gut microbes, and sepsis. Gut Microbes.

[69] Johnston J, Walker W (1983). Experimental model of ischemic bowel necrosis. The role of platelet-activating factor and endotoxin. J Pathol.

[70] Borthakur A, Bhattacharyya S, Alrefai WA, Tobacman JK, Ramaswamy K, Dudeja PK (2010). Platelet-activating factor-induced NF-kappaB activation and IL-8 production in intestinal epithelial cells are Bcl10-dependent. Inflamm Bowel Dis.

[71] Casillan AJ, Gonzalez NC, Johnson JS, Steiner DR, Wood JG (2003). Mesenteric microvascular inflammatory responses to systemic hypoxia are mediated by PAF and LTB4. J Appl Physiol (1985).

[72] Touloukian RJ, Posch JN, Spencer R (1972). The pathogenesis of ischemic gastroenterocolitis of the neonate: selective gut mucosal ischemia in asphyxiated neonatal piglets. J Pediatr Surg.

[73] Hansbrough F, Priebe CJ, Bornside GH (1983). Pathogenesis of early necrotizing enterocolitis in the hypoxic neonatal dog. Am J Surg.

[74] Karna P, Senagore A, Chou CC (1986). Comparison of the effect of asphyxia, hypoxia, and acidosis on intestinal blood flow and O2 uptake in newborn piglets. Pediatr Res.

[75] Barlow B, Santulli TV, Heird WC (1974). An experimental study of acute neonatal enterocolitis--the importance of breast milk. J Pediatr Surg.

[76] Yu Y, Zhang X, Hong S (2014). The expression of platelet-activating factor receptor modulates the cisplatin sensitivity of ovarian cancer cells: a novel target for combination therapy. Br J Cancer.

[77] Caplan MS, Sun XM, Hsueh W (1990). Hypoxia causes ischemic bowel necrosis in rats: the role of platelet-activating factor (PAF-acether). Gastroenterology.

[78] Souza DG, Cara DC, Cassali GD (2000). Effects of the PAF receptor antagonist UK74505 on local and remote reperfusion injuries following ischaemia of the superior mesenteric artery in the rat. Br J Pharmacol.

[79] Souza DG, Pinho V, Soares AC, Shimizu T, Ishii S, Teixeira MM (2003). Role of PAF receptors during intestinal ischemia and reperfusion injury. A comparative study between PAF receptor-deficient mice and PAF receptor antagonist treatment. Br J Pharmacol.

[80] Cho SX, Berger PJ, Nold-Petry CA, Nold MF (2016). The immunological landscape in necrotising enterocolitis. Expert Rev Mol Med.

[81] Qu XW, Rozenfeld RA, Huang W, Sun X, Tan XD, Hsueh W (1999). Roles of nitric oxide synthases in platelet-activating factor-induced intestinal necrosis in rats. Crit Care Med.

[82] Buchwalow IB, Podzuweit T, Bocker W (2002). Vascular smooth muscle and nitric oxide synthase. FASEB J.

[83] Qu XW, Thaete LG, Rozenfeld RA, Zhu Y, De Plaen IG, Caplan MS, Hsueh W (2005). Tetrahydrobiopterin prevents platelet-activating factor-induced intestinal hypoperfusion and necrosis: role of neuronal nitric oxide synthase. Crit Care Med.

[84] Tan XD, Chang H, Qu XW, Caplan M, Gonzalez-Crussi F, Hsueh W (2000). Platelet-activating factor increases mucosal permeability in rat intestine via tyrosine phosphorylation of E-cadherin. Br J Pharmacol.

[85] Nepali PR, Burboa PC, Lillo MA (2023). Endothelial mechanisms for inactivation of inflammation-induced hyperpermeability. Am J Physiol Heart Circ Physiol.

[86] Ravisankar S, Tatum R, Garg PM, Herco M, Shekhawat PS, Chen YH (2018). Necrotizing enterocolitis leads to disruption of tight junctions and increase in gut permeability in a mouse model. BMC Pediatr.

[87] Gribar SC, Sodhi CP, Richardson WM (2009). Reciprocal expression and signaling of TLR4 and TLR9 in the pathogenesis and treatment of necrotizing enterocolitis. J Immunol.

[88] Good M, Siggers RH, Sodhi CP (2012). Amniotic fluid inhibits Toll-like receptor 4 signaling in the fetal and neonatal intestinal epithelium. Proc Natl Acad Sci U S A.

[89] Claud EC, Lu J, Wang XQ (2008). Platelet-activating factor-induced chloride channel activation is associated with intracellular acidosis and apoptosis of intestinal epithelial cells. Am J Physiol Gastrointest Liver Physiol.

[90] Deng Z, Zhao Y, Ma Z (2021). Pathophysiological role of ion channels and transporters in gastrointestinal mucosal diseases. Cell Mol Life Sci.

[91] Lu J, Caplan MS, Saraf AP, Li D, Adler L, Liu X, Jilling T (2004). Platelet-activating factor-induced apoptosis is blocked by Bcl-2 in rat intestinal epithelial cells. Am J Physiol Gastrointest Liver Physiol.

[92] Fan TJ, Han LH, Cong RS, Liang J (2005). Caspase family proteases and apoptosis. Acta Biochim Biophys Sin (Shanghai).

[93] Czabotar PE, Garcia-Saez AJ (2023). Mechanisms of BCL-2 family proteins in mitochondrial apoptosis. Nat Rev Mol Cell Biol.

[94] Ola MS, Nawaz M, Ahsan H (2011). Role of Bcl-2 family proteins and caspases in the regulation of apoptosis. Mol Cell Biochem.

[95] Syed MK, Al Faqeeh AA, Saeed N (2021). Surgical versus medical management of necrotizing enterocolitis with and without intestinal perforation: a retrospective chart review. Cureus.

[96] Zozaya C, García González I, Avila-Alvarez A (2020). Incidence, treatment, and outcome trends of necrotizing enterocolitis in preterm infants: a multicenter cohort study. Front Pediatr.

[97] Liu Y, Qiao L, Wu X, Jiang Z, Hao X (2022). Predictive factors for the surgical treatment of necrotizing enterocolitis in preterm infants: a single-center retrospective study. BMC Pediatr.

[98] Blackwood BP, Hunter CJ, Grabowski J (2017). Variability in antibiotic regimens for surgical necrotizing enterocolitis highlights the need for new guidelines. Surg Infect (Larchmt).

[99] Dierikx TH, Deianova N, Groen J (2022). Association between duration of early empiric antibiotics and necrotizing enterocolitis and late-onset sepsis in preterm infants: a multicenter cohort study. Eur J Pediatr.

[100] Fu C, Sun W, Wang X, Zhu X (2023). Human breast milk: a promising treatment for necrotizing enterocolitis. Early Hum Dev.

[101] Dasgupta S, Jain SK (2017). Protective effects of amniotic fluid in the setting of necrotizing enterocolitis. Pediatr Res.

[102] Afrazi A, Sodhi CP, Richardson W, Neal M, Good M, Siggers R, Hackam DJ (2011). New insights into the pathogenesis and treatment of necrotizing enterocolitis: toll-like receptors and beyond. Pediatr Res.

[103] O'Connell JS, Lee C, Farhat N, Antounians L, Zani A, Li B, Pierro A (2021). Administration of extracellular vesicles derived from human amniotic fluid stem cells: a new treatment for necrotizing enterocolitis. Pediatr Surg Int.

[104] Li B, Lee C, Chuslip S (2021). Intestinal epithelial tight junctions and permeability can be rescued through the regulation of endoplasmic reticulum stress by amniotic fluid stem cells during necrotizing enterocolitis. FASEB J.

[105] de Kroon RR, de Baat T, Senger S, van Weissenbruch MM (2022). Amniotic fluid: a perspective on promising advances in the prevention and treatment of necrotizing enterocolitis. Front Pediatr.

[106] Jain SK, Baggerman EW, Mohankumar K, Namachivayam K, Jagadeeswaran R, Reyes VE, Maheshwari A (2014). Amniotic fluid-borne hepatocyte growth factor protects rat pups against experimental necrotizing enterocolitis. Am J Physiol Gastrointest Liver Physiol.

[107] de Jong JC, Ijssennagger N, van Mil SW (2021). Breast milk nutrients driving intestinal epithelial layer maturation via Wnt and Notch signaling: implications for necrotizing enterocolitis. Biochim Biophys Acta Mol Basis Dis.

[108] Tongviratskool C, Pongsakul N, Kanaprach P (2022). How does human milk protect against necrotizing enterocolitis (NEC)? Targeted validation and time-course analysis of 35 gene responses as NEC-signature in fetal intestinal epithelial cells. OMICS J Integr Biol.

[109] Dermyshi E, Wang Y, Yan C, Hong W, Qiu G, Gong X, Zhang T (2017). The "Golden age" of probiotics: a systematic review and meta-analysis of randomized and observational studies in preterm infants. Neonatology.

[110] Tayman C, Uckan D, Kilic E (2011). Mesenchymal stem cell therapy in necrotizing enterocolitis: a rat study. Pediatr Res.

[111] Demers-Mathieu V, Huston RK, Markell AM, McCulley EA, Martin RL, Spooner M, Dallas DC (2019). Differences in maternal immunoglobulins within mother's own breast milk and donor breast milk and across digestion in preterm infants. Nutrients.

[112] Dasgupta S, Arya S, Choudhary S, Jain SK (2016). Amniotic fluid: Source of trophic factors for the developing intestine. World J Gastrointest Pathophysiol.

[113] Chen W, Wang X, Yan X, Yu Z, Zhang J, Han S (2020). The emerging role of exosomes in the pathogenesis, prognosis and treatment of necrotizing enterocolitis. Am J Transl Res.

[114] Markel TA, Crisostomo PR, Lahm T, Novotny NM, Rescorla FJ, Tector J, Meldrum DR (2008). Stem cells as a potential future treatment of pediatric intestinal disorders. J Pediatr Surg.

[115] Cacho NT, Parker LA, Neu J (2017). Necrotizing enterocolitis and human milk feeding: a systematic review. Clin Perinatol.

[116] Mao F, Wu Y, Tang X (2017). Exosomes derived from human umbilical cord mesenchymal stem cells relieve inflammatory bowel disease in mice. Biomed Res Int.

[117] McCulloh CJ, Olson JK, Wang Y, Zhou Y, Tengberg NH, Deshpande S, Besner GE (2018). Treatment of experimental necrotizing enterocolitis with stem cell-derived exosomes. J Pediatr Surg.

[118] Li B, Lee C, O'Connell JS (2020). Activation of Wnt signaling by amniotic fluid stem cell-derived extracellular vesicles attenuates intestinal injury in experimental necrotizing enterocolitis. Cell Death Dis.

[119] Hu X, Zhang R, Liang H (2023). Comparison and investigation of exosomes from human amniotic fluid stem cells and human breast milk in alleviating neonatal necrotizing enterocolitis. Stem Cell Rev Rep.

[120] Rager TM, Olson JK, Zhou Y, Wang Y, Besner GE (2016). Exosomes secreted from bone marrow-derived mesenchymal stem cells protect the intestines from experimental necrotizing enterocolitis. J Pediatr Surg.

[121] Li B, Lee C, Cadete M (2022). Amniotic fluid stem cell administration can prevent epithelial injury from necrotizing enterocolitis. Pediatr Res.

[122] Pisano C, Besner GE (2019). Potential role of stem cells in disease prevention based on a murine model of experimental necrotizing enterocolitis. J Pediatr Surg.

[123] Nolan LS, Parks OB, Good M (2019). A review of the immunomodulating components of maternal breast milk and protection against necrotizing enterocolitis. Nutrients.

[124] Lu J, Pierce M, Franklin A, Jilling T, Stafforini DM, Caplan M (2010). Dual roles of endogenous platelet-activating factor acetylhydrolase in a murine model of necrotizing enterocolitis. Pediatr Res.

[125] Sönmezgöz E, Takci S, Gül A (2021). Ursodeoxycholic acid protects neonatal rats from necrotizing enterocolitis: a biochemical, histopathological, and immunohistochemical study. J Matern Fetal Neonatal Med.

[126] Sami AS, Frazer LC, Miller CM, Singh DK, Clodfelter LG, Orgel KA, Good M (2023). The role of human milk nutrients in preventing necrotizing enterocolitis. Front Pediatr.

[127] Jensen ML, Thymann T, Cilieborg MS (2014). Antibiotics modulate intestinal immunity and prevent necrotizing enterocolitis in preterm neonatal piglets. Am J Physiol Gastrointest Liver Physiol.

[128] Bury RG, Tudehope D (2000). Enteral antibiotics for preventing necrotising enterocolitis in low birthweight or preterm infants. Cochrane Database Syst Rev.

[129] Li Y, Shen RL, Ayede AI (2020). Early use of antibiotics is associated with a lower incidence of necrotizing enterocolitis in preterm, very low birth weight infants: the NEOMUNE-NeoNutriNet cohort study. J Pediatr.

[130] Jiang YN, Muk T, Stensballe A, Nguyen DN, Sangild PT, Jiang PP (2020). Early protein markers of necrotizing enterocolitis in plasma of preterm pigs exposed to antibiotics. Front Immunol.

[131] Smith MJ, Boutzoukas A, Autmizguine J (2021). Antibiotic safety and effectiveness in premature infants with complicated intra-abdominal infections. Pediatr Infect Dis J.

[132] Luo LJ, Li X, Yang KD, Lu JY, Li LQ (2015). Broad-spectrum antibiotic plus metronidazole may not prevent the deterioration of necrotizing enterocolitis from stage II to III in full-term and near-term infants: a propensity score-matched cohort study. Medicine (Baltimore).

[133] Warner BB, Deych E, Zhou Y (2016). Gut bacteria dysbiosis and necrotising enterocolitis in very low birthweight infants: a prospective case-control study. Lancet.

[134] Nguyen DN, Fuglsang E, Jiang P (2016). Oral antibiotics increase blood neutrophil maturation and reduce bacteremia and necrotizing enterocolitis in the immediate postnatal period of preterm pigs. Innate Immun.

[135] Esaiassen E, Fjalstad JW, Juvet LK, van den Anker JN, Klingenberg C (2017). Antibiotic exposure in neonates and early adverse outcomes: a systematic review and meta-analysis. J Antimicrob Chemother.

[136] Agakidou E, Agakidis C, Gika H, Sarafidis K (2020). Emerging biomarkers for prediction and early diagnosis of necrotizing enterocolitis in the era of metabolomics and proteomics. Front Pediatr.

[137] Abdulkadir B, Nelson A, Skeath T (2016). Stool bacterial load in preterm infants with necrotising enterocolitis. Early Hum Dev.

